# Evaluating the potential of anti-dsRNA antibodies as an alternative viral sensing tool in encephalitides of different species

**DOI:** 10.3389/fvets.2025.1540437

**Published:** 2025-03-21

**Authors:** Madeleine de le Roi, Hannah Gerhards, Adnan Fayyad, Mathias Boelke, Stefanie Christine Becker, Asisa Volz, Ingo Gerhauser, Wolfgang Baumgärtner, Christina Puff

**Affiliations:** ^1^Department of Pathology, University of Veterinary Medicine Hannover, Hannover, Germany; ^2^Department of Veterinary Medicine, Faculty of Agriculture and Veterinary Medicine, An-Najah National University, Nablus, Palestine; ^3^Institute of Parasitology, University of Veterinary Medicine Hannover, Hannover, Germany; ^4^Institute of Virology, University of Veterinary Medicine Hannover, Hannover, Germany

**Keywords:** formalin-fixed and paraffin-embedded (FFPE) tissue, immunohistochemistry, negative-sense single-stranded RNA viruses, non-suppurative meningoencephalitis, positive-sense single-stranded RNA viruses, virus detection

## Abstract

Although laboratory methods have advanced, the cause of many encephalitides is still unknown. Molecular methods like multiplex PCR and microarrays are considered to be often less sensitive than Next Generation Sequencing, whereas the latter is time-consuming and costly. These analyses require appropriate tissue preparations and are more difficult to perform on formalin-fixed, paraffin-embedded (FFPE) tissues. Anti-double-stranded RNA (dsRNA) antibodies could potentially identify virus infections independently of the viral genome and can be applied to FFPE material. This study examined the applicability of monoclonal anti-dsRNA antibodies by immunohistochemistry to confirm encephalitides caused by different RNA viruses and comparing the findings with those obtained using monoclonal and polyclonal virus-specific antibodies. The viruses studied included negative-sense (Borna disease virus 1, BoDV-1; canine distemper virus, CDV; Rift Valley fever virus, RVFV) and positive-sense single stranded RNA viruses (severe acute respiratory disease syndrome coronavirus 2, SARS-CoV-2; tick-borne encephalitis virus, TBEV; Theiler’s murine encephalomyelitis virus, TMEV). Interestingly, dsRNA was detected in both infected and non-infected animals and inconsistently co-localized to BoDV-1, TBEV, and TMEV antigen. Strict co-localization was lacking in CDV, SARS-CoV-2 and RVFV. Despite the co-localization of dsRNA with virus antigen for some RNA viruses, anti-dsRNA antibodies were unreliable as markers for unknown virus infections. Future studies should explore the upstream components of the immune response, including the interferon signaling cascade to assess their potential as effective virus-sensing tool.

## 1 Introduction

Despite progress in diagnostic techniques and laboratory methods, a considerable number of encephalitides with unknown cause, sharing similar histopathological pattern, remains unidentified (1–3). Viruses are particularly known to induce non-suppurative inflammatory processes in different organ systems, including the central nervous system (CNS) (4). In clinic, the diagnostic process for encephalitis begins with a general and neurological examination based on the patient’s medical history, followed by an analysis of the cerebrospinal fluid (CSF) (5). The CSF is tested for glucose, protein and cell content, and various polymerase chain reaction (PCR) methods are used to detect potential pathogens (5). Since pathogen detection is highly dependent on the timing of CSF collection, it is also recommended to assess antibodies against common encephalitis pathogens in both CSF and serum using enzyme immunoassays (5). The next step in the diagnostic process is neuroimaging, preferably with magnetic resonance imaging (5). In approximately 30–60% of the cases, the etiology remains undetermined, and brain biopsies or post mortem tissue samples are further analyzed in pathology (6). The first step involves a histopathological evaluation of the tissue. The distribution and extent of inflammation, the type of inflammatory cells and specific cytological features such as inclusion bodies provide crucial diagnostic insights (5). Particularly, non-suppurative encephalitis often suggests a viral cause (7). In cases, suspicious of having a viral etiology, tissues will then be tested for known viruses using conventional methods including immunohistochemistry (IHC), various PCR techniques or *in situ* hybridization (ISH) (3, 8). Instead of singleplex PCR tests, multiplex PCR and microarray are utilized, as they enable the detection of different viruses in a single run (9). Microarrays also provide the advantage of analyzing the pathogen’s genotype (5). While multiplex PCR and microarrays are often considered less sensitive than real-time PCR, all three methods exhibit comparably high specificity (10, 11). Even after further examination and exclusion of all common well-known viruses, a large proportion of cases remains undetermined (12–14). In a previous study investigating non-suppurative meningoencephalitides in dogs and cats, a viral cause could not be demonstrated in the majority of cases (4). This might be related to the fact that most conventional methods are virus-specific and may not detect viral variants or newly emerging viruses. Next Generation Sequencing (NGS) provides a sequence-independent approach that offers the possibility to identify so far overlooked viral variants or newly emerging viruses. Nevertheless, the generated data must possess a certain degree of similarity to already known viruses in order to achieve a match by comparing the obtained results with data available (8, 15). Additionally, this method is very cost-intensive and time-consuming (16–18). The success in discovering new viruses relies not only on the methods employed but also on the material utilized for the investigations. Formalin-fixed, paraffin-embedded (FFPE) specimens are the most commonly used material in pathology and offer several advantages in the field of virus discovery (19, 20). To access viral antigens and nucleic acids in FFPE specimens for investigating potential viral causes, various pre-treatment techniques can be employed to retrieve viral structures. Furthermore, FFPE material can be stored at room temperature (RT) for extended periods, allowing retrospective analyses of past disease outbreaks even several years after sampling (19, 21, 22). In this context, a previous study from 2023 identified the newly discovered rustrela virus (RusV) as the cause of encephalitis in captive lions from the 1980s (23). The presence of RusV RNA and antigen in the FFPE tissues of these lions was detected using the panRusV-2 a broadly reactive quantitative reverse transcription PCR (qRT-PCR) assay, ISH and IHC (23). In addition, a part of the RusV genome was determined by Sanger sequencing (23). Moreover, the maintained tissue morphology of FFPE samples facilitates the examination of viral structures in association with histopathological lesions and enables the investigation of the cell tropism (24–27). Although the process of formalin fixation and embedding in paraffin can have a negative impact on the integrity of these structures, e.g., the degradation of the nucleic acids, the development of new screening methods that are easily applicable to stored FFPE material may help to overcome the frequent unavailability of native or properly frozen organ samples (19, 20, 28–32). Immunohistochemical detection of double-stranded ribonucleic acids (dsRNA) in cases with suspected viral etiology of unknown origin represents a promising approach to enhance the identification of previously unknown viruses, due to its broad applicability to commonly used FFPE tissue samples. Antibodies against dsRNA are considered to represent a pan-viral detection method, as their use allows virus infections to be identified independently of the genomic constitution of the causative virus (33, 34). Because dsRNA molecules are synthesized independently of the virus genome, the detection of dsRNA is considered to be virus-independent and not limited to already known viral proteins or sequences, as in the case of antibodies, probes or primers. The generation of viral dsRNA is determined by the viral genome and distinct replication strategies employed by various virus groups. Single-stranded RNA (ss RNA) viruses use their genome as a template to synthesize complementary negative-sense and positive-sense RNA strands, respectively. During the replication of positive-sense single-stranded RNA viruses (+ss RNA viruses), the complementary negative-sense RNA strand functions as a template for generating new positive-sense RNA genomes. In contrast, negative-sense single-stranded RNA viruses (-ss RNA viruses) must initially convert their genome into complementary positive-strand RNA before protein synthesis can take place. In both cases, the reverse-oriented copies occur simultaneously with the genomic RNA, and accidental hybridization of both strands can result in the formation of dsRNA intermediates (15, 34–39). However, previous studies indicated that significantly smaller amounts of dsRNA intermediates are generated during -ss RNA virus infections compared to infections caused by +ss RNA viruses (34, 37). The genome of dsRNA viruses is the primary source of dsRNA in infections caused by these viruses. Although this group of viruses specifically attempts to conceal dsRNA from the immune system detection by replicating within viroplasms, these dsRNA molecules can still be found in the cytoplasm of the infected cells. DNA viruses generate dsRNA by overlapping transcription (15, 34–39). The present study investigated the applicability of antibodies directed against dsRNA as a sensing tool for virus infections in encephalitides of suspected viral origin, regardless of the specific virus or host species. Therefore, this study examined cases of encephalitis with known causes including Borna disease virus 1 (BoDV-1), canine distemper virus (CDV) and Rift Valley fever virus (RVFV) as representatives of -ss RNA viruses as well as severe acute respiratory syndrome coronavirus 2 (SARS-CoV-2), tick-borne encephalitis virus (TBEV) and Theiler’s murine encephalomyelitis virus (TMEV) as examples of +ss RNA viruses.

## 2 Materials and methods

### 2.1 Animals and tissues

In this study, FFPE samples from 45 animals were analyzed, including virus-infected (*n* = 23/45) and control animals (*n* = 22/45), representing horses, dogs and mice. Additionally, both *in vitro*, with the vaccine strain Onderstepoort (Ond) infected canine histiocytic sarcoma cells [DH82 cells, European Collection of Cell Cultures (ECACC), Salisbury, GB] and non-infected DH82 cells were also examined. The experimentally infected animals and cells originated from previous studies. Detailed information regarding the experimental design can be found in the references mentioned in Table 1.

**Table 1:** Overview of investigated animals, including the type of viral genome, virus, species, etiology, animal experimentation permission number and corresponding reference.

Viral genome	Virus	Species	Etiology	Permission number	References
-ss RNA	BoDV-1	Horse	Naturally occuring BoDV-1 infection (*n* = 3)		
			Control (*n* = 3)		
	CDV	Dog	Naturally occuring CDV infection (*n* = 5)		(71, 72)
			Control (*n* = 4)		
		DH82 cells	Persistently CDV Ond infected (*n* = 1)		
			Control (*n* = 1)		
	RVFV	C57BL/6 mouse	Experimental RVFV infection; 7, 9, 11 dpi (*n* = 4)	33.19–42,502–04-19/3323	(73)
			Control (*n* = 4)		
+ss RNA	SARS-CoV-2	B6.Cg-Tg (K18-ACE2)2Prlmn/J mouse	Experimental SARS-CoV-2 infection, 6 dpi (*n* = 3)	33.8–42,502–04-20/3440	Unpublished data
			Control (*n* = 3)		
		C57BL/6 mouse	Control (*n* = 1)		
	TBEV	C57BL/6 mouse	Experimental TBEV infection, 6 dpi (*n* = 4)	33.9–42,502–04-18/2804	(47)
			Control (*n* = 4)		
	TMEV	SJL/JCrHs mouse	Experimental TMEV infection, 7 dpi (*n* = 4)	17/2418	(74)
			Control (*n* = 3)		

-ss RNA, negative-sense single-stranded RNA; +ss RNA, positive-sense single-stranded RNA; BoDV-1, Borna disease virus 1; CDV, canine distemper virus; control, no significant histological lesions; DH82, canine histiocytic sarcoma cells; dpi, days post infection; n, number; RVFV, Rift Valley fever virus; SARS-CoV-2, severe acute respiratory syndrome coronavirus 2; TBEV, tick-borne encephalitis virus; TMEV, Theiler’s murine encephalomyelitis virus.

Regarding SARS-CoV-2, transgenic B6.Cg-Tg (K18-ACE2)2Prlmn/J (K18*hACE2*) mice expressing human angiotensin-converting enzyme 2 (hACE2) were intranasally infected with SARS-CoV-2 [BavPat1/2020 strain, 5×10^4^ tissue culture infectious dose (TCID) 50%] and euthanized 6 days post infection. For each virus group, the same localizations of the brain of virus-infected and non-infected animals were examined but differed slightly between the different viruses. In detail, the investigations on BoDV-1 in infected and control horses included cerebral cortex and the hippocampus. From two CDV-infected dogs and one control dog, only cerebrum was available. From the remaining six infected and non-infected dogs, from experimentally RVFV-, SARS-CoV-2-, TBEV-, and TMEV-infected mice as well as from mock-infected control mice the entire brain, including cerebrum, cerebellum and brain stem, was available for examination. In addition, experimentally Umatilla virus (UMAV) infected quail fibroblasts isolated from a quail with fibrosarcoma (ATCC, University Boulevard Manassas, VA, United States) have been used as a positive control for dsRNA expression.

### 2.2 Histopathology and immunohistochemistry

Organ samples were collected during necropsy and fixed in 10% neutral buffered formalin for at least 24 h (40). After trimming, samples were embedded in paraffin and cut into 2–4 μm thick sections (40). Afterwards, sections were mounted on SuperFrost®Plus slides (Glasbearbeitungswerke GmbH & Co. KG, Braunschweig, Germany). For histopathological examination, sections were routinely stained with hematoxylin and eosin (HE) (41). Immunohistochemistry was used to investigate the expression of viral antigens and dsRNA and was performed as previously described (42). The staining protocol for the anti-dsRNA antibodies used was optimized through a multi-step procedure including various pretreatments (e.g., heat induced antigen retrieval, proteinase K pretreatment), antibody dilutions and test systems (e.g., Dako EnVision+ System-HRP labeled polymer). In addition to infected and non-infected tissues of the respective species, UMAV-infected quail fibroblasts were used as a reliable and robust positive control during the establishment process of the anti-dsRNA staining. Table 2 provides detailed information about the primary antibodies. Viral antigens were detected with following antibodies: monoclonal anti-BoDV-1 antibody Bo18 (43), monoclonal anti-CDV nucleoprotein antibody D110 (44), polyclonal anti-RVFV antibody S24Np (45), polyclonal anti-SARS-CoV-2 spike protein (SARS-CoV-2 S) antibody (46), polyclonal anti-TBEV antibody (47) and polyclonal anti-TMEV antibody (48). Immunohistochemical detection of dsRNA was achieved using the following monoclonal antibodies: anti-dsRNA antibody J2, anti-dsRNA antibody K1 and anti-dsRNA antibody 9D5 (42). Briefly, deparaffinization and rehydration was followed by inhibition of endogenous peroxidase by incubating the sections in 85% ethanol with 0.5% hydrogen peroxide for 30 min at RT (49). Antigen retrieval was applied according to the primary antibody (Table 2). For the detection of CDV, SARS-CoV-2 S, J2 and K1, heat-induced epitope retrieval was conducted in the respective buffer solution in a microwave at 800 W for 20 min and subsequent cooling-down for 20 min at RT. For TBEV and anti-dsRNA 9D5, proteolytic-induced epitope retrieval was achieved by incubation with Proteinase K [1,000 mL phosphate buffered saline (PBS) admixed with 0.3 mg Proteinase K (Roche Diagnostics, Mannheim Germany)] for 7 min at RT. No antigen retrieval was performed for the screening of BoDV-1, RVFV and TMEV. Afterwards, sections were incubated for 30 min at RT with either inactivated normal goat serum or normal rabbit serum (diluted 1:5 in PBS, respectively), depending on the secondary antibody, to reduce unspecific binding reactions. Afterwards, primary antibodies were applied in their respective concentration (Table 2) and sections were incubated over night at 4°C. For negative controls, primary antibodies were replaced either by Balb/c mouse ascitic fluid (diluted 1:1,000 in PBS, Cedarlane^®^, biologo, Kronshagen, Germany), by rabbit serum (diluted 1:3,000 in PBS, Sigma-Aldrich Chemie GmbH, Tauffkirchen, Germany) or by applying sheep serum (diluted 1:3,000 in PBS, serum of sheep from the Clinic for Swine, Small Ruminants and Forensic Medicine, University of Veterinary Medicine Hannover, Germany) depending on the host species of the respective antibody. Thereafter, sections were incubated with secondary biotinylated goat-anti-mouse antibody (VECTOR^®^, Biozol Diagnostica Vertrieb GmbH, Eching, Germany), goat-anti-rabbit antibody (VECTOR^®^, Biozol Diagnostica Vertrieb GmbH, Eching, Germany) or rabbit-anti-sheep antibody (VECTOR^®^, Biozol Diagnostica Vertrieb GmbH, Eching, Germany), diluted 1:200 in PBS, respectively for 30 min at RT. After washing twice with PBS, sections were incubated with avidin–biotin-peroxidase complex (ABC, Vectastain ABC Kit Standard, Vector Laboratories, Burlingame, CA, United States) for 30 min at RT. In experimentally RVFV-, SARS-CoV-2-, TBEV- and TMEV-infected mice, as well as in the respective control animals, the Mouse-on-Mouse Polymer IHC Kit (Abcam Limited, Cambridge, United Kingdom) was applied when using anti-dsRNA antibodies (50). For this, the tissue sections were covered with the blocking reagent for 30 min at RT prior to incubation with the primary antibody. Instead of using ABC, the sections were subsequently incubated with the horse radish peroxidase polymer detector reagent for 15 min at RT. Signal detection was achieved by the application of 3,3′-diaminobenzidine tetrahydrochloride (0.05%, Sigma Aldrich Chemie GmbH, Darmstadt, Germany) with 0.03% hydrogen peroxide for 5 min at RT. Counterstaining was performed with Mayer’s hematoxylin (Roth C. GmbH & Co KG, Karlsruhe, Germany). Due to the varying intensities of anti-dsRNA staining, a semiquantitative rather than a quantitative grading was used to ensure better sustainable interpretation of the results. For analysis, the number of immunopositive cells expressing viral antigen and dsRNA was graded semiquantitatively in serial sections based on total tissue area as follows: - = no immunopositive cells (no immunoreactivity), + = 1 to 25% immunopositive cells (minimal number of immunopositive cells), ++ = 25–50% immunopositive cells (low number of immunopositive cells), +++ = 50–75% immunopositive cells (moderate number of immunopositive cells), and ++++ = 75–100% immunopositive cells (high number of immunopositive cells). The number of immunopositive cells was determined for each section.

**Table 2:** Overview of used primary antibodies, their target structure, clonality, host species, dilution, applied antigen retrieval method, detection system and antibody source.

Target structure	Clone	Clonality, species	Dilution	Antigen retrieval	Detection system	Source of antibody
BoDV-1 nucleoprotein	Bo18	Mc, mouse	1:500	none	ABC	Kindly provided by Friedrich-Loeffler Institute, Greifswald-Insel Riems, Germany
CDV nucleo-protein	D110	Mc, mouse	1:1,000	Citrate buffer, microwave (800 W, 20 min)	ABC	Kindly provided by Prof. Dr. A. Zurbriggen, University of Bern, Switzerland
RVFV nucleo-protein	S24Np	Pc, sheep	1:50,000	None	ABC	Kindly provided by Friedrich-Loeffler Institute, Greifswald-Insel Riems, Germany
SARS-CoV-2- spike protein	SARS-CoV-2 S	Pc, rabbit	1:4,000	EDTA buffer, microwave (800 W, 20 min)	ABC	Sino Biological, Peking, China
TBEV	TBEV serum	Pc, rabbit	1:2,000	Proteinase K	ABC	Kindly provided by Center for Virology, Medical University of Vienna, Austria
TMEV	TMEV serum	Pc, rabbit	1:2,000	None	ABC	(75)
dsRNA	J2	Mc, mouse	1:300	Citrate EDTA buffer, microwave (800 W, 20 min)	Mouse-on-Mouse Polymer IHC Kit*	Jena Bioscience, Jena, Germany
					ABC**	
dsRNA	K1	Mc, mouse	1:150	Citrate buffer, microwave (800 W, 20 min)	Mouse-on-Mouse Polymer IHC Kit*	Jena Bioscience, Jena, Germany
					ABC**	
dsRNA	9D5	Mc, mouse	1:100	Proteinase K	Mouse-on-Mouse Polymer IHC Kit*	Absolute antibody, Wilton, UK
					ABC**	

ABC, avidin-biotin complex; BoDV-1, Borna disease virus 1; CDV, canine distemper virus; dsRNA, double-stranded RNA; EDTA, ethylenediaminetetraacetic acid; mc, monoclonal; pc, polyclonal; RVFV, Rift Valley fever virus; SARS-CoV-2, severe acute respiratory syndrome coronavirus 2; TBEV, tick-borne encephalitis virus; TMEV, Theiler’s murine encephalomyelitis virus; *, used for murine tissues; **, used for all non-murine tissues.

## 3 Results

### 3.1 Histopathology

**Figure 1:**
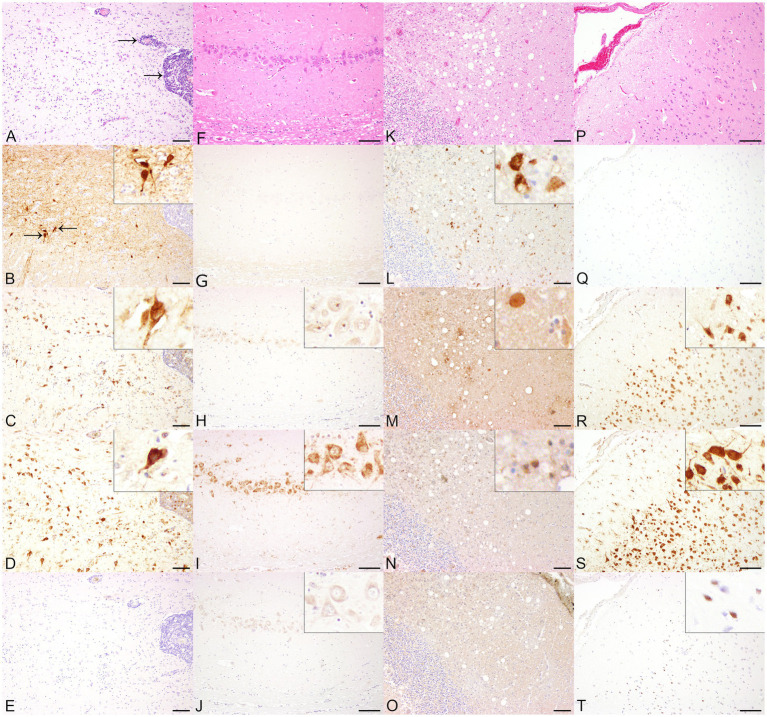
Histopathological and immunohistochemical findings in animals infected with single-stranded RNA viruses with negative polarity (−ss RNA viruses), including Borna disease virus 1 (BoDV-1, **A–E**) and canine distemper virus (CDV, **K–O**) and and non-infected control animals (horses **F–J**, dogs **P–T**) in serial sections. **(A)** The cerebrum of a BoDV-1-infected horse displayed a multifocal, perivascular, lympho-histiocytic encephalitis (arrows). Hematoxylin and eosin (HE). **(B)** BoDV-1-specific antigen was demonstrated predominantly within cerebral cortical neurons. Insert: Cerebral cortical neurons expressed BoDV-1-specific antigen within cytoplasm and nucleus. Immunohistochemistry (IHC), higher magnification. Both anti-double-stranded RNA (dsRNA) antibodies J2 **(C)** and K1 **(D)** displayed a cytoplasmic and intranuclear granular staining of cerebral cortical neurons. Inserts: Immunopositive staining was found cytoplasmically and intranuclearly within neurons. IHC, higher magnification. **(E)** No immunopositive reaction was obtained by using 9D5. IHC. Bars indicate 100 μm. **(F)** No significant histopathological lesions were present in the hippocampus of a non-infected horse. HE. Although the cerebrum of the horse was tested negative for BoDV-1-specific antigen **(G)**, dsRNA was detected cytoplasmically and/or intranuclearly in hippocampal neurons by using J2 **(H)**, K1 **(I)**, and 9D5 **(J)**. Inserts: Immunopositive signal for dsRNA in neurons of the hippocampus at higher magnification. IHC. Bars indicate 100 μm. **(K)** The cerebellum of a CDV-infected dog with subacute lesions showed demyelination of the white matter. HE. **(L)** Multifocally, glial cells expressed CDV nucleoprotein. IHC. The immunohistochemical investigation for the expression of dsRNA in the cerebellum of a CDV-infected dog revealed a minimal number of cytoplasmically immunopositive glial cells using J2 **(M)**, K1 **(N)**, but remained negative using 9D5 **(O)**. IHC. Bars indicate 100 μm. **(P)** No histopathological lesions were found in the cerebral cortex of a control dog. HE. **(Q)** Although the screening for CDV nucleoprotein remained negative, dsRNA expression was observed in cerebral cortical neurons by using J2 **(R)**, K1 **(S)**, and in glial cells using 9D5 **(T)**. Inserts: DsRNA-positive cortical neurons and glial cells at higher magnification. IHC. Bars indicate 100 μm.

**Figure 2:**
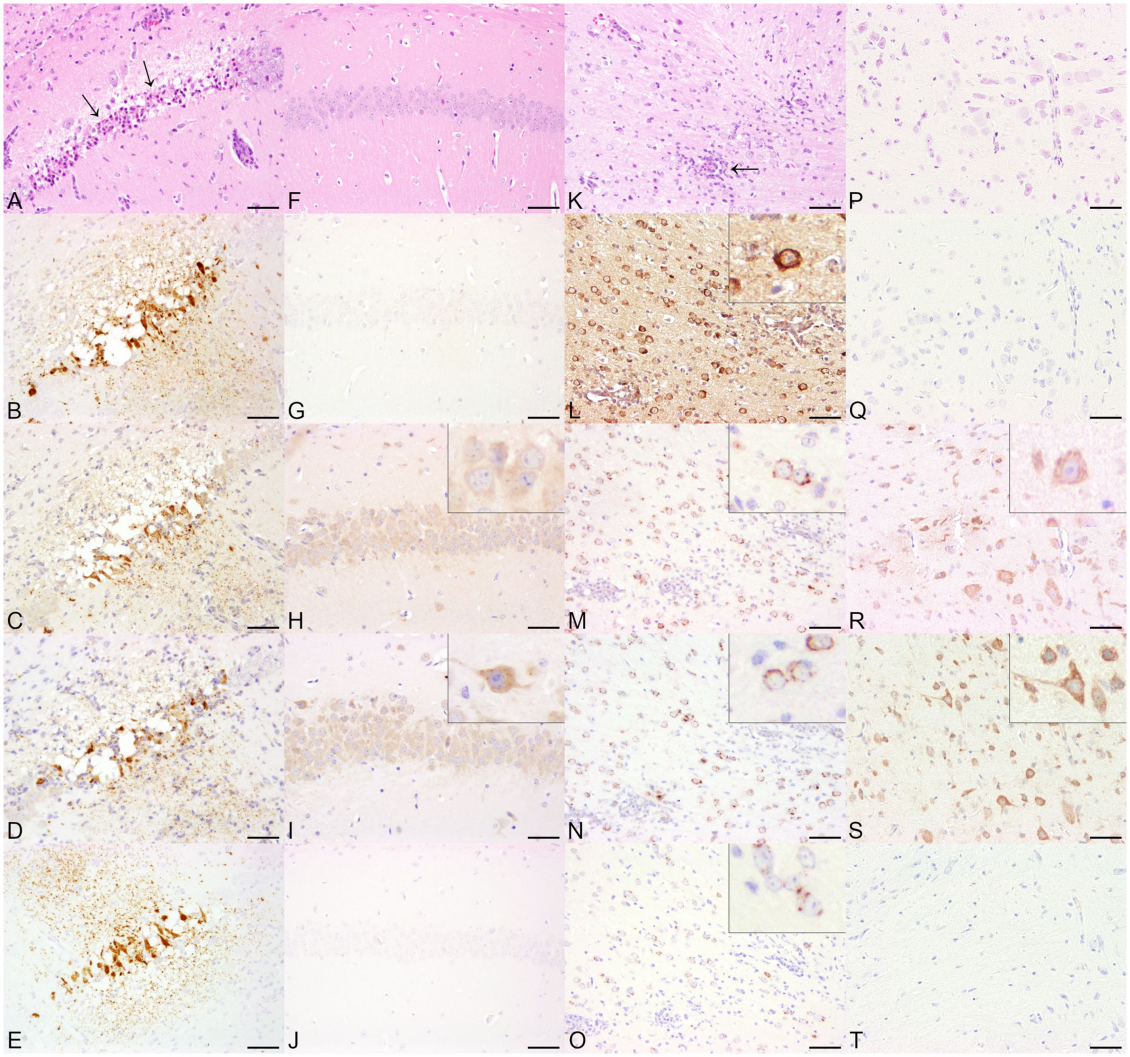
Histopathological and immunohistochemical findings in animals infected with single-stranded RNA viruses with positive polarity (+ss RNA viruses), including Theiler’s murine encephalomyelitis virus (TMEV, **A–E**) and tick-borne encephalitis virus (TBEV, **K–O**) and non-infected control animals (TMEV, **F–J**, TBEV, **P–T**) in serial sections. **(A)** The cerebrum of a TMEV-infected mouse displayed multifocal neuronal necrosis (arrows) and multifocal neuronal loss within the hippocampus. Hematoxylin and eosin (HE). **(B)** Necrotic neurons and the neuropil were positive for TMEV antigen. Immunohistochemistry (IHC). The localizations that tested positive for viral antigen also expressed double-stranded RNA (dsRNA) using J2 **(C)**, K1 **(D),** and 9D5 **(E)**. IHC. Bars indicate 50 μm. **(F)** The histological examination of the hippocampus of a control mouse revealed no significant histopathological alterations. HE. **(G)** The brain of the mouse was tested negative for the expression of TMEV-specific antigen. Multifocal to coalescing, cells of the hippocampus tested positive for dsRNA using J2 **(H)** and K1 **(I)**. Inserts: The J2 signal consisted of a homogeneous cytoplasmic reaction, whereas the immunopositive pattern by K1 showed a predominantly granular cytoplasmic distribution. **(J)** In contrast, no immunolabeling was found by the application of 9D5. IHC. Bars indicate 50 μm. **(K)** The cerebrum of a TBEV-infected mouse showed a multifocal, lympho-histiocytic, perivascular encephalitis (arrow). HE. **(L)** TBEV antigen was demonstrated multifocally within the cerebrum. Insert: TBEV-specific antigen was predominantly observed within cerebral cortical perikarya. IHC, higher magnification. Immunopositive signals for dsRNA were achieved by the application of J2 **(M)**, K1 **(N)**, and 9D5 **(O)**. Inserts: For all three anti-dsRNA antibodies, the immunopositive signal was characterized by a perinuclear, granular signal within cerebral cortical neurons. IHC, higher magnification. **(P)** The cerebral cortex of a control mouse was histologically unremarkable. HE. Despite the lack of TBEV-specific antigen **(Q)**, the application of anti-dsRNA antibodies J2 **(R)** and K1 **(S)** resulted in multifocal immunopositive cerebral cortical neurons. Inserts: In contrast to J2, which displayed a homogeneous cytoplasmic signal, the K1 reaction exhibited a granular appearance. **(T)** No immunoreaction was found using 9D5. IHC. Bars indicate 50 μm.

BoDV-1-infected horses showed a multifocal, perivascular, lympho-histiocytic encephalitis in the cerebrum, predominantly affecting the hippocampus, but also occurring in the cerebral cortex (Figure 1A). Histopathological lesions of CDV-infected dogs comprised moderate, multifocal, cerebellar demyelination (Figure 1K) accompanied by multifocal glial cell proliferation with gemistocytes, gitter cells, and eosinophilic inclusion bodies in astrocytes as well as lymphocytic, inconsistently perivascular encephalitis. RVFV-infected mice displayed multifocal necrosis of cerebral neurons, especially in the hippocampus (Supplementary Figure S1A), accompanied by lympho-histiocytic meningoencephalitis. Main histopathological lesions of the brain of experimentally SARS-CoV-2-, TBEV-, and TMEV-infected mice consisted of a multifocal, lympho-histiocytic, perivascular meningoencephalitis (Supplementary Figure S2A; Figures 2A,K). Brain tissue of TMEV-infected mice additionally exhibited perilesional gliosis, multifocal neuronal necrosis and multifocal neuronal loss limited to the hippocampus (Figure 2A). Furthermore, TBEV-infected mice showed a concentrically arranged gliosis around vessels. Neither the brain of non-infected control animals (Figures 1F,P, 2F,P; Supplementary Figures S1F, S2F) nor non-infected DH82 cells displayed significant histopathological lesions.

### 3.2 Comparison of the distribution and correlation of immunohistochemical expression of viral antigen and dsRNA

As expected, immunohistochemical investigation for the presence of viral antigen revealed immunopositive reactions in all virus-infected animals and CDV-infected DH82 cells. Furthermore, immunolabeling for viral antigen remained negative in non-infected control animals and non-infected DH82 cells. In contrast, the application of anti-dsRNA antibodies yielded positive immunoreactivity in 43 of 45 animals and in cell pellets included in this study, regardless of whether they were infected or not. It is important to note that the morphological appearance of the immunopositive reactions for dsRNA showed considerable variability, ranging from no signal to a homogenous or granular reaction pattern. The signal was highly variable among the different virus groups and the three different anti-dsRNA antibodies. Additionally, the subcellular distribution of the signals varied, with immunoreactions either in the nucleus or in the cytoplasm. The following sections provide a detailed description of the results for each virus group. In addition, Table 3 provides an overview of the semiquantitative evaluation of the expression of viral antigen and dsRNA.

**Table 3:** Overview of investigated animals including type of viral genome, virus, species, etiology and immunohistochemical scoring results of the expression of viral antigen and double-stranded RNA (dsRNA).

Viral genome	Virus	Species	Etiology	Immunhistochemical expression of			
				Viral antigen	dsRNA J2	dsRNA K1	dsRNA 9D5
-ss RNA	BoDV-1	Horse	Naturally occuring BoDV-1 infection (*n* = 3)	+ to ++	++ to +++	++ to +++	−
			Control (*n* = 3)	−	+ to ++	+ to +++	+
	CDV	Dog	Naturally occuring CDV infection (*n* = 5)	+	− to ++	− to ++	−
			Control (n = 4)	−	+	++	+++
		DH82 cells	Persistently CDV-infected (*n* = 1)	+	+	+	+
			Control (*n* = 1)	−	+ to ++	++	− to +
	RVFV	C57BL/6 mouse	Experimental RVFV infection; 7, 9, 11 dpi (*n* = 4)	++ to +++	++	++ to +++	−
			Control (*n* = 4)	−	++	++	−
+ss RNA	SARS-CoV-2	B6.Cg-Tg (K18-ACE2)2Prlmn/J mouse	Experimental SARS-CoV-2 infection, 6 dpi (*n* = 3)	+ to ++	++	++	−
			Control (*n* = 3)	−	− to ++	+ to +++	−
		C57BL/6 mouse	Control (*n* = 1)	−	+	++	−
	TBEV	C57BL/6 mouse	Experimental TBEV infection, 6 dpi (*n* = 4)	++	++ to +++	+++	+
			Control (*n* = 4)	−	+	+++	−
	TMEV	SJL/JCrHs mouse	Experimental TMEV infection, 7 dpi (*n* = 4)	+	++	++	+
			Control (*n* = 3)	−	++	++	−

BoDV-1, Borna disease virus 1; CDV, canine distemper virus; control, no significant histological lesions; DH82, canine histiocytic sarcoma cells; dpi, days post infection; dsRNA, double-stranded RNA; n, number; RVFV, Rift Valley fever virus; SARS-CoV-2, severe acute respiratory syndrome coronavirus 2; TBEV, tick-borne encephalitis virus; TMEV, Theiler’s murine encephalomyelitis virus; −, no immunopositive cells; + = 1 to 25% immunopositive cells; ++ = 25–50% immunopositive cells; +++ = 50–75% immunopositive cells; ++++ = 75–100% immunopositive cells.

### 3.3 BoDV-1

BoDV-1 nucleoprotein was detected in the cerebrum of all three naturally BoDV-1 infected horses, predominantly localized in the cytoplasm and nuclei of hippocampal neurons, as well as multifocally in cerebral cortical neurons (Figure 1B), astrocytes, ependymal cells and oligodendrocytes. The number of immunopositive cells varied from minimal to low. Immunolabeling for dsRNA demonstrated the presence of dsRNA in the cerebrum of BoDV-1-infected horses using the antibodies J2 and K1. Compared to BoDV-1 antigen, semiquantitative evaluation showed an increased number of immunopositive cells for dsRNA, ranging from mild to moderate. Both J2 (Figure 1C) and K1 (Figure 1D) exhibited a granular cytoplasmic and intranuclear signal, which was found predominantly in the same regions that tested positive for viral antigen but also in perivascular areas and localizations lacking viral antigen expression. In addition, one of the three animals also showed a homogeneous immunopositive signal for dsRNA in cortical neurons when J2 was used. No immunolabeled cells were detected using the antibody 9D5 (Figure 1E). Although the brains of three non-infected control horses were tested negative for viral antigen (Figure 1G), the application of the three anti-dsRNA antibodies revealed minimal to moderate numbers of dsRNA-positive neurons in the cerebral cortex and hippocampus of all three animals. The application of J2 predominantly resulted in a homogeneous, inconsistently intranuclear immunopositive signal (Figure 1H), while K1 staining was characterized by a granular cytoplasmic reaction (Figure 1I). In contrast, the signal of 9D5 appeared as a homogeneous cytoplasmic reaction (Figure 1J).

### 3.4 CDV

Immunohistochemical expression of CDV nucleoprotein ranged from minimal to low numbers of immunopositive cells, detected multifocally in the grey matter. CDV antigen was predominantly observed in cortical neurons and astrocytes, and to a lesser extent within meningeal, ependymal and vascular endothelial cells, as well as in glial cells of the cerebellar white matter (Figure 1L) and perivascularly in all CDV-infected dogs. Additionally, more than 90% of CDV-infected DH82 cells expressed CDV nucleoprotein. Minimal to low numbers of dsRNA-immunopositive cells were observed in three CDV-infected dogs as well as in persistently CDV-infected DH82 cells using J2 and K1. In the brains of CDV-infected dogs, both antibodies exhibited a homogeneous immunopositive signal (Figures 1M,N) predominantly in regions that tested negative for CDV nucleoprotein. The signal was diffusely distributed in the cytoplasm of cerebral cortical neurons and Purkinje cells. Immunohistochemical staining for dsRNA using the 9D5 antibody remained negative in these three CDV-infected dogs (Figure 1O). Furthermore, two CDV-infected dogs tested negative for dsRNA by using all three anti-dsRNA antibodies. In contrast, brain of all four non-infected dogs as well as non-infected DH82 cells lacking CDV nucleoprotein expression displayed immunopositive signals for dsRNA when J2 and K1 were applied. In addition, two of these dogs and non-infected DH82 cells also expressed dsRNA using 9D5. For all three antibodies, immunopositive reactions were located cytoplasmically in cerebral cortical neurons (Figures 1R,S), Purkinje cells, hippocampal neurons, and even inconsistently within cerebral cortical glial cells (Figure 1T). Morphological appearance of immunopositive reactions exhibited a wide range from diffusely homogeneous to granular.

### 3.5 RVFV

RVFV-specific nucleoprotein expression was observed in all four RVFV-infected mice. Multifocally, low to moderate numbers of neurons expressing viral antigen were found within the cerebral cortex, hippocampus (Supplementary Figure S1B) and cerebellum. Labeling of dsRNA with J2 and K1 produced diffuse, homogeneous cytoplasmic staining in Purkinje cells, cerebellar and hippocampal neurons (Supplementary Figures S1C,D). In all control animals lacking viral antigen expression (Supplementary Figure S1G), the dsRNA signal obtained with J2 and K1 (Supplementary Figures S1H,I) was similar to that seen in RVFV-infected animals (Supplementary Figures S1C,D). Additionally, the distribution pattern of dsRNA was identical to that observed in RVFV-infected mice. Immunohistochemical investigation using the antibody 9D5 was negative in all RVFV-infected animals (Supplementary Figure S1E) and non-infected control mice (Supplementary Figure S1J). However, an immunopositive signal for 9D5 was repeatedly observed in several stainings of the positive control.

### 3.6 SARS-CoV-2

Minimal to low numbers of SARS-CoV-2 S were predominantly located multifocally in the cytoplasm of cerebral cortical neurons (Supplementary Figure S2B), as well as in brain stem and hippocampal neurons, but were absent in the cerebellum of experimentally SARS-CoV-2-infected K18 mice. J2 and K1 displayed predominantly a mild, nearly diffusely distributed, homogeneous cytoplasmic immunoreactivity in cerebral cortical neurons (Supplementary Figures S2C,D), brain stem neurons, and Purkinje cells. Occasionally, both J2 and K1 staining resulted in a homogeneous signal in brain stem neurons and a granular signal in hippocampal and cerebral cortical neurons. DsRNA expression by using 9D5 was negative (Supplementary Figure S2E). SARS-CoV-2 S was absent in all four control K18 mice (Supplementary Figure S2G). However, minimal to moderate numbers of dsRNA-positive cells were observed multifocally to coalescing in all non-infected control mice using K1, and in three of four non-infected control mice using J2. While J2 staining was characterized by a homogeneous signal in hippocampal and cerebral cortical neurons (Supplementary Figure S2H), staining with K1 showed considerable differences: In two control mice, the signal was similar to that observed with J2, while the other two control mice exhibited a granular signal in cerebral cortical neurons and hippocampal neurons when using K1 (Supplementary Figure S2I). As already observed in SARS-CoV-2-infected mice, screening with 9D5 remained negative in all non-infected control mice (Supplementary Figure S2J).

### 3.7 TBEV

In all TBEV infected mice, low numbers of cells expressing viral antigen were distributed widely throughout the cerebrum and were particularly noticed multifocally within cerebral cortical neuronal perikarya (Figure 2L), in neurons of the brain stem and hippocampus, as well as occasionally within Purkinje cells. The three antibodies sensing dsRNA exhibited a distribution pattern similar to that of the viral antigen, although the number of dsRNA-positive cells was increased for J2 and K1 compared to the number of cells expressing TBEV antigen. In contrast, the number of dsRNA-positive cells detected by 9D5 was lower than the number of TBEV antigen-positive cells. The immunopositive signal for dsRNA in TBEV-infected mice appeared as a cytoplasmic granular reaction for all three antibodies (Figures 2M,O). Minimal to moderate numbers of immunopositive cells for dsRNA were also observed in all non-infected control mice using J2 and K1, while no immunoreactivity was present using 9D5 (Figure 2T). The investigation with J2 resulted in a homogeneous cytoplasmic staining of cerebral cortical neurons (Figure 2R). The immunopositive signal of K1 in non-infected control mice appeared partially granular (Figure 2S) and was additionally found in Purkinje cells and in neurons of the brain stem of all four control animals.

### 3.8 TMEV

Except for single immunopositive signals within the surrounding neuropil of the lateral ventricle, TMEV-specific antigen was almost limited to necrotic neurons and the adjacent neuropil of the hippocampus (Figure 2B) in all TMEV-infected mice. Immunopositive reactions for the expression of dsRNA were demonstrated with all three antibodies. While only minimal numbers of cells expressing viral antigen and dsRNA were detected by 9D5, low numbers of dsRNA-positive cells were found by applying J2 and K1. All three dsRNA antibodies exhibited a cytoplasmic, granular staining particularly in necrotic neurons of the hippocampus (Figures 2C–E) as well as in the surrounding neuropil. The signal distribution was similar to that observed for TMEV antigen. In addition, a multifocal to coalescing signal was noticed in cerebral cortical neurons as well as in Purkinje cells and neurons of the brain stem by the application of J2 and K1. While the J2 signal in these localizations appeared homogeneous, dsRNA staining with K1 was characterized by a granular signal in these areas lacking viral antigen. Immunohistochemical investigation of non-infected control animals lacking viral antigen (Figure 2G) revealed dsRNA expression in all three mice. Using J2 and K1, low numbers of dsRNA-immunopositive cells were detected multifocally in cerebral cortical neurons, Purkinje cells and neurons of the hippocampus. As already noted in TBEV-infected animals, the J2 signal was homogeneous (Figure 2H), while the K1 signal was predominantly granular (Figure 2I). 9D5 revealed no immunopositive reaction in any of the three control mice (Figure 2J).

## 4 Discussion

Immunohistochemical investigation of brain tissue from animals displaying virus-induced encephalitis (*n* = 23) and non-infected control animals (*n* = 22) revealed immunopositive reactions for dsRNA in 43 of 45 animals investigated, including dsRNA detection in 21 virus-infected animals and all 22 non-infected animals. In addition, the expression of dsRNA was also detected in DH82 cells, independently of whether the cells were CDV-infected or non-infected. The immunohistochemical analysis of dsRNA yielded negative results in two CDV-infected dogs by the application of all three anti-dsRNA antibodies. This lack of detectable dsRNA is likely due to several factors. Variations in viral load and the timing of sampling may contribute to the negative results. Histopathological findings, such as demyelination and lymphocytic perivascular inflammation, suggest a subacute to chronic inflammatory response (51). As the disease progresses, the detectable antigen decreases, which could explain the absence of dsRNA signals (52). Second, CDV, like other -ss RNA viruses, produces only small amounts of dsRNA and the detection of dsRNA relies on ongoing viral replication (34). If the viral load is low or if replication-defective viruses are present, this could explain the negative results observed. Additionally, the post-mortem processing of tissue samples collected over the past 30 years may also influence the outcome. Some samples may have undergone prolonged fixation in improperly concentrated or unbuffered formalin, which is known to fragment nucleic acids and mask epitopes (20). Furthermore, RNA degradation could have occurred during the post-mortem interval before fixation and extended storage of FFPE tissues may have led to further degradation, all of which could hinder the detection of dsRNA (53, 54). For measles virus, another member of the *Paramyxoviridae* family, dsRNA formation has already been detected in experimentally infected cells (34). Interestingly, the intensity of the immunopositive signal was considerably weaker than that of +ss RNA viruses (34). It has been proposed that the C protein, which modulates innate immune responses, may contribute to reduced dsRNA production during measles virus infection (34). This was further supported by demonstrating a considerable decrease in the amount of dsRNA in cells infected with a wild type measles virus compared to cells infected with a mutant measles virus with an induced knockout of the C protein (55). Similarly, for CDV, the expression of the C protein is known to result in significantly fewer dsRNA-positive cells, which helps the virus evading the host’s innate immune system (56). Attenuated variants of CDV, often with a defective C protein, produce excessive amounts of defective RNA, which aggregates into dsRNA (56). Given this, a stronger dsRNA signal would have been expected in the CDV Ond infected DH82 cells. The morphological appearance of the immunopositive anti-dsRNA signal differed considerably, being present either as diffuse homogeneous cytoplasmic, granular cytoplasmic, intranuclear, or as an extracellular signal. In three CDV-infected dogs as well as in non-infected controls, cerebral cortical neurons and Purkinje cells showed an almost diffuse immunopositive signal for dsRNA. Surprisingly, the dsRNA signal in CDV-infected dogs and CDV-infected DH82 cells was homogeneously distributed within the cytoplasm, whereas in non-infected dogs and DH82 cells, the immunopositive signal appeared predominantly granular. The homogeneous cytoplasmic signal observed in CDV-infected animals is likely nonspecific. CDV replicates in the cytoplasm through RNA-dependent RNA polymerase (57). This process takes place in membrane-less organelles, enriched with high levels of N- and P-proteins (58). Given this mode of replication, a granular dsRNA signal would be anticipated, reflecting the formation of large cytoplasmic aggregates, rather than the homogeneous signal that was observed. The use of J2 and K1 in BoDV-1-infected horses resulted in a convincing granular reaction co-localized to viral antigen. A similar signal was detected in non-infected horses using K1, occurring in areas where virus antigen expression would typically be expected. Furthermore, the immunopositive dsRNA reaction was observed in both the cytoplasm and the nucleus, although BoDV-1 replicates within the nucleus (59). This finding is somewhat surprising, as –ssRNA viruses like BoDV-1 are known to produce relatively low amounts of dsRNA during viral replication (34, 37). Nevertheless, the usefulness of dsRNA antibodies in BoDV-1 appears to be limited, as similar positive signals were also observed in non-infected horses. In contrast, it was shown that dsRNA was successfully detected in cells infected with Nyamanini virus, a member of the order *Mononegavirales* and therefore related to BoDV-1 (34, 60, 61). The study by Son et al. (34) also used a panel of three anti-dsRNA antibodies, including J2 and 9D5, while Thomsen et al. (62) applied J2 and 9D5, and Richardson et al. (8) used J2 and K1. These studies differed in terms of protocols and, in some cases, the antibody manufacturers. One notable difference is that Son et al. (34) and Thomsen et al. (62) detected a strong 9D5 signal, unlike the present study. Additionally, the present study and the investigation by Thomsen et al. (62) detected dsRNA signals in control animals, suggesting possible non-specific binding. While the current study examined both +ss RNA and –ss RNA viruses, only Son et al. (34) investigated both types of viruses. Similar to our findings a weaker dsRNA signal was detected for –ss RNA viruses compared to +ss RNA viruses. The present study and the investigations by Thomsen et al. (62) and Richardson et al. (8) included tissues from naturally infected animals and cell lines from various animal species. In contrast, Son et al. (34) used cell lines, fixed with 10% formalin or 3.7% paraformaldehyde, and viruses that likely underwent multiple *in vitro* passages. It is important to note that viral attenuation in cell culture may influence the expression of virulence factors and dsRNA production, as demonstrated in studies of measles and CDV (55, 56). In all RVFV-infected mice as well as in all non-infected control mice, a predominantly homogeneous signal, almost diffusely distributed throughout the brain, was detected. It remains questionable whether the weak signal represents dsRNA or a non-specific background staining. Nonetheless, the staining pattern does not allow any differentiation between RVFV-infected and non-infected mice. As already described for RVFV, the application of J2 and K1 resulted in cytoplasmic homogeneous signals in the brain of SARS-CoV-2-infected K18 mice. However, in contrast to RVFV, the application of K1 resulted in a more intense, inconsistently granular signal in non-infected control K18 mice. On the other hand, no difference in J2 signaling was detected in infected and non-infected K18 mice. Coronaviruses have developed strategies to circumvent the innate immune system, which are partially based on the fact that dsRNA recognition by host factors initiates an antiviral response (63–67). Nevertheless, detectable amounts of dsRNA were demonstrated in experimentally SARS-CoV-2-infected cells (67). TBEV and TMEV represent the only two viruses with a granular signal co-localized to viral antigen in all infected animals using all three anti-dsRNA antibodies. Based on the morphology of the signal and the positivity of all three antibodies, it was assumed that this represents a true positive signal for the expression of dsRNA. However, even in these cases it must be noted that in non-infected control mice, staining with J2 resulted in a homogeneous cytoplasmic signal, while K1 displayed a partially granular reaction. The few to no signals observed with the 9D5 antibody, compared to the positive immunoreactivity seen with J2 and K1 in non-infected control mice could be explained by the fact that the low number of positive cells observed by J2 and K1 antibodies may reflect an unspecific signal. In contrast, the absence of a similar number of 9D5-positive cells could indicate the higher specificity of this antibody. Granular immunopositive anti-dsRNA reactions were exclusively observed in BoDV-1-infected horses and TBEV- and TMEV-infected mice, suggesting that these antibodies might indeed bind to dsRNA in the context of viral infections. However, the presence of similar signals in non-infected control animals raises questions about the specificity of these antibodies in the broader context of viral encephalitides. This finding is consistent with previous studies that have highlighted the limitations of dsRNA antibodies in distinguishing between infected and non-infected states, particularly in diseases caused by -ss RNA viruses (34, 37). In these previous studies, dsRNA was demonstrated mainly in mammalian FFPE cell pellets, e.g., infected with Coxsackie virus (+ss RNA virus), measles virus and influenza A virus (−ss RNA viruses), and minute virus of mice, lymphocytic choriomeningitis virus, vaccinia virus and adenovirus (DNA viruses) (8, 34, 37). Similarly, dsRNA could be successfully visualized in neonatal mouse FFPE organ samples and human neonatal FFPE heart samples infected with Coxsackie virus (8). It therefore remains undetermined whether species-specific differences may also represent factors influencing dsRNA expression. Similarly, in own studies analyzing Usutu virus-infected birds and rustrela virus-infected lions, immunopositive signals were observed in different organs of infected animals and virus-negative control animals (22, 42).The hypothesis that the virus and animal species have a potential impact on dsRNA immunoreactivity is supported by the considerable differences observed in the results from BoDV-1-infected horses and CDV-infected dogs, despite both viruses belonging to the order *Mononegavirales* (61, 68). Similar observations have been made in SARS-CoV-2 infections: While previous investigations on the demonstration of dsRNA in experimentally infected cells were successful, the present results indicate that dsRNA expression was lacking in SARS-CoV-2 infected brains of K18 mice (63). However, it is important to consider whether the species-specific differences might also be partly due to the use of different detection kits, including ABC kit and Mouse-on-Mouse Polymer IHC kit for immunohistochemical staining of dsRNA in the different species. Furthermore, it remains unclear whether the anti-dsRNA antibodies sensed exclusively viral dsRNA intermediates. It is assumed that the anti-dsRNA antibodies recognize viral dsRNA intermediates regardless of the nucleotide sequence, but with a nucleotide length of at least 50 base pairs (bp) (8). This would also reduce the possibility of the detection of endogenous dsRNA, as these molecules are usually shorter than 50 bp (8, 69). In order to be used as a reliable screening tool for potential virus infections, these antibodies should exhibit a higher specificity for viral dsRNA. For example, virus-specific epitopes like the 5′-triphosphate end could be incorporated into the recognition process of the antibody (70). Additionally, the sensitivity of the antibody should be increased so that even small amounts of dsRNA, such as those produced during the replication of –ssRNA viruses, can be reliably detected in the future.

## 5 Conclusion

The investigation of the expression of dsRNA in animals and cells infected with various –ss RNA viruses and +ss RNA viruses has produced some notable findings. Specifically, the study observed the co-localization of anti-dsRNA signals with viral antigens in BoDV-1-infected horses. The latter is a -ss RNA virus with only low levels of dsRNA expression during viral replication. Additionally, granular immunopositive signals in TBEV- and TMEV-infected animals, co-localizing with viral antigens using all three anti-dsRNA antibodies, suggest the potential of these antibodies as markers for viral infection. However, the usability of anti-dsRNA antibodies as an early detection marker of viral infections was considered very limited due to immunopositive signals in non-infected control animals and cells. While the reactions in controls are disturbing, they also present a point for discussion and further refinement. These findings emphasize the need to enhance the specificity of anti-dsRNA antibodies, which could help to distinguish better infected from non-infected tissues in future studies.

## Data Availability

The original contributions presented in the study are included in the article/Supplementary material, further inquiries can be directed to the corresponding authors.
