# Supplementary material for: Evaluating the potential of anti-dsRNA antibodies as an alternative viral sensing tool in encephalitides of different species
Source: Front Vet Sci. 2025 Mar 21;12:1540437. doi: 10.3389/fvets.2025.1540437 (PMC11969456; doi:10.3389/fvets.2025.1540437)
Supplement: Supplementary file 1 [file Data_Sheet_1.PDF]

Evaluating the potential of anti-dsRNA antibodies as an alternative viral sensing tool in encephalitides of different species

Madeleine de le Roi[1,†], Hannah Gerhards[1,†], Adnan Fayyad[1,2], Mathias Boelke[3], Stefanie Becker[3], Asisa Volz[4], Ingo Gerhauser[1], Wolfgang Baumgärtner[1,*], Christina Puff[1]

[1]Department of Pathology, University of Veterinary Medicine Hannover, Germany

[2]Department of Veterinary Medicine, Faculty of Agriculture and Veterinary Medicine, An-Najah National University, Nablus, Palestine

[3]Institute of Parasitology, University of Veterinary Medicine Hannover, Germany

[4]Institute of Virology, University of Veterinary Medicine Hannover, Germany

[†]These authors contributed equally to this work

Supplementary Material

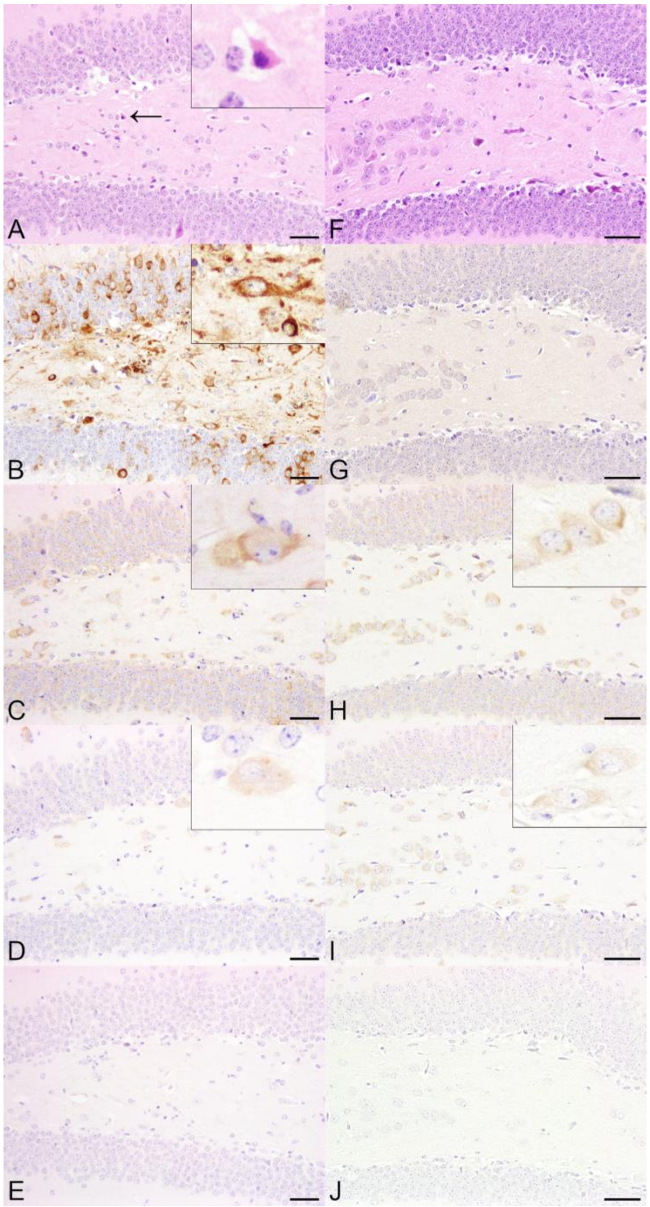

Supplementary Figure 1. Histopathological and immunohistochemical findings in C57BL/6 mice infected with Rift Valley fever virus (RVFV, **A-E**) a single-stranded RNA viruses with negative polarity (-ss RNA viruses) and non-infected control animals (**F-J**) in serial sections. (**A**) Hippocampal lesions of a RVFV-infected mouse consisted of multifocal neuronal necrosis (arrow). Insert: Necrotic neuron displaying an eosinophilic, shrunken and triangular morphology. Hematoxylin and eosin (HE), higher magnification. (**B**) RVFV-specific antigen was found in neurons of the hippocampus. Insert: Demonstration of RVFV antigen within the cytoplasm of a neuron. Immunohistochemistry (IHC), higher magnification. The staining for double-stranded RNA (dsRNA) by using J2 (**C**) and K1 (**D**) resulted in a homogeneous cytoplasmic reaction in neurons of the

hippocampus. Inserts: The cytoplasm of neurons stained homogeneously brown. IHC, higher magnification. (**E**) By applying 9D5, no immunoreactivity was observed. IHC. Bars indicate 50 µm. (**F**) Histopathological examination of the hippocampus of a control mouse revealed no significant alterations. HE. (**G**) Despite the absence of RVFV-specific antigen, the application of the anti-dsRNA antibodies J2 (**H**) and K1 (**I**) resulted in multifocal cytoplasmic immunopositive reactions in neuronal cells. Inserts: The immunopositive signal for J2 and K1 was characterized by a homogeneously distributed signal within the cytoplasm of neurons of the hippocampus. (**J**) The screening for dsRNA using 9D5 was negative. IHC. Bars indicate 50µm.

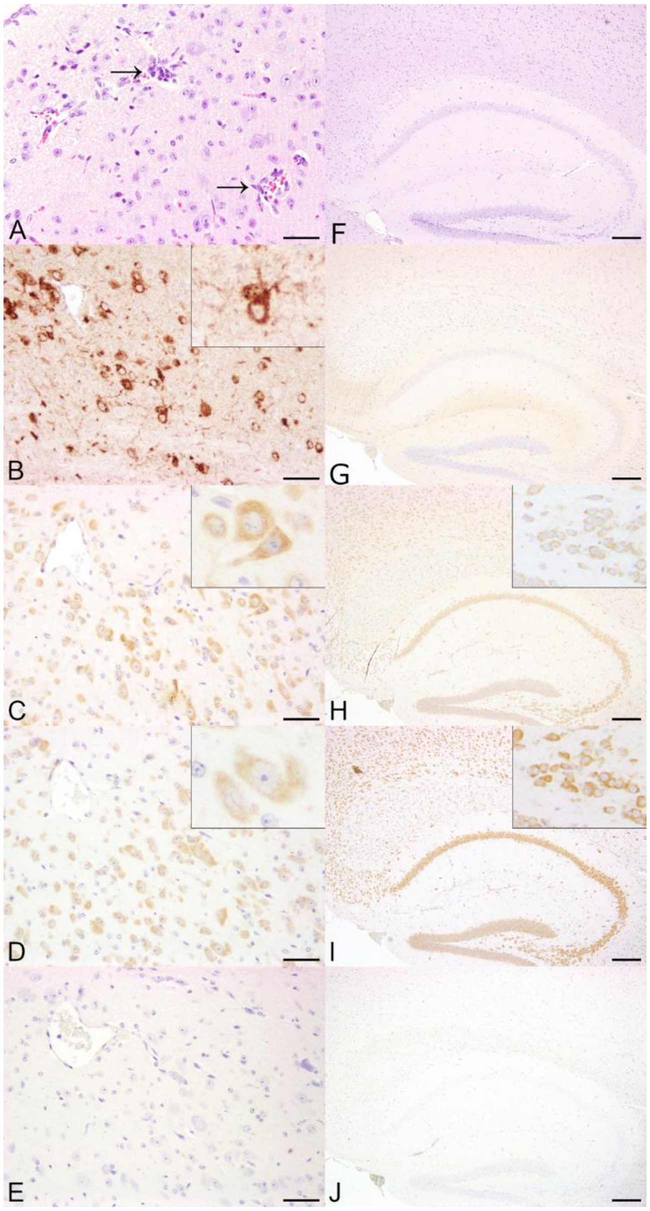

Supplementary Figure 2. Histopathological and immunohistochemical findings in B6.Cg-Tg(K18-ACE2)2Prlmn/J mice infected with severe acute respiratory syndrome coronavirus 2 (SARS-CoV-2, **A**-**E**), a single-stranded RNA viruses with positive polarity (+ss RNA viruses) and non-infected control animals (**F**-**J**) in serial sections. (**A**) Histopathological examination of a SARS-CoV-2-infected mouse revealed a multifocal, lympho-histiocytic, perivascular encephalitis (arrows). Hematoxylin and eosin (HE). (**B**) SARS-CoV-2 spike protein was predominantly found cytoplasmically in cerebral cortical neurons. Immunohistochemistry (IHC). Investigation of double-stranded RNA (dsRNA), illustrated by the use of J2 (**C**) and K1 (**D**) revealed immunopositive reactions adjacent to areas tested positive for viral antigen. Inserts: Immunopositive reactions of J2 and K1 were characterized by a homogeneous, cytoplasmic staining of neurons. IHC, higher magnification. (**E**) In contrast to J2 and K1, 9D5 did not display any immunopositive staining. IHC. (**F**) Light microscopy of the cerebrum of a control K18 mouse revealed no histopathological lesions. HE. (**G**) No SARS-CoV-2 spike protein expression was observed within the cerebrum. IHC.

Immunohistochemical investigation for dsRNA yielded multifocal immunopositive neurons in the cerebral cortex and the hippocampus using J2 (**H**) and K1 (**I**). Inserts: While the J2 signal appeared homogeneous in the cytoplasm, the K1 signal was characterized by granular cytoplasmic staining. (**J**) The staining with 9D5 did not result in immunopositive reactions. IHC. Bars indicate 200µm.
